# A Backwards Approach to Bariatric Surgery: The Perioperative Approach Used in a Woman with Situs Inversus Totalis Undergoing a Laparoscopic Sleeve Gastrectomy

**DOI:** 10.7759/cureus.3464

**Published:** 2018-10-18

**Authors:** Yadira Villalvazo, Candice M Jensen

**Affiliations:** 1 Miscellaneous, University of Arizona College of Medicine-Tucson, Tucson, USA; 2 Surgery, Yuma Regional Medical Center, Yuma, USA

**Keywords:** situs inversus totalis, laparoscopic sleeve gastrectomy, bariatric surgery, obesity

## Abstract

Situs inversus totalis is a rare congenital condition where organs are mirrored across the sagittal plane of the body. In the absence of associated comorbidities, most people have normal health and lifespan. Challenges with mirrored image anatomy arise when needing an operative procedure involving the intraabdominal organs. There are few reported cases in the literature of laparoscopic surgery in patients with situs inversus, with even fewer in the field of bariatric surgery. Obesity and obesity-related comorbidities continue to increase in our society, and bariatric surgery is a treatment option for weight loss. We report the perioperative approach used in a 59-year-old obese woman with confirmed situs inversus totalis undergoing laparoscopic sleeve gastrectomy.

## Introduction

Situs inversus is a congenital anomaly with the organs positioned in mirror image. It is estimated to be one out of 8,000 to 25,000 births and follows an autosomal recessive inheritance pattern [1]. A person can have both intrathoracic and intraabdominal compartment involvement, which is defined as situs inversus totalis, as with our patient, or just involvement of one compartment [1]. While most individuals with situs inversus totalis have normal health and normal life expectancy, people can have associated respiratory, cardiovascular, and digestive anomalies [1]. Thus, pre-procedural evaluation including abdominal X-ray and computed tomography (CT), upper gastrointestinal series (UGI), upper endoscopy, cardiac echocardiogram and pulmonary function analysis was done for this patient.

The prevalence of obesity, defined as a body mass index (BMI) > 30 kg/m^2^, and obesity-related co-morbidities, continues to rise and bariatric surgery has become a viable weight loss option for patients. It has gained popularity over the years and has been shown as a successful long-term treatment for morbid obesity [2]. In the United States, metabolic and bariatric procedures increased nearly 10% from 2015 to 2016 [2]. The laparoscopic sleeve gastrectomy (LSG) has become one of the most commonly performed bariatric procedures, and 125,318 LSGs were performed in 2016 in the United States, accounting for 58% of all the bariatric procedures that year [2]. The LSG entails resecting the greater curvature and fundus of the stomach promoting weight loss through restricting gastric volume and distensibility, as well as through hormonal modification. After the procedure, Ghrelin, a hormone that stimulates appetite, is reduced secondary to the removal of the gastric fundus [3]. Additionally, Glucagon-like peptide, a hormone that increases insulin secretion which stimulates glucose metabolism, reduction of hunger and satiety, is increased [3]. Criteria to undergo surgical management of severe obesity, as set by the National Institutes of Health Consensus Development Panel in 1991, include: BMI > 40 kg/m^2^ with or without any associated comorbidities, BMI between 35 and 39.9 kg/m^2^ with at least one weight-related comorbidity (including but not limited to type 2 diabetes, obstructive sleep apnea, hypertension, obesity-hypoventilation syndrome, non-alcoholic fatty liver disease, gastroesophageal reflux and debilitating arthritis), and BMI between 30 and 34.9 kg/m^2^ with uncontrolled type 2 diabetes or metabolic syndrome [4]. A patient who undergoes LSG can expect to lose on average 60.5% of excess body weight in five years and achieve a BMI of about 30.2 kg/m^2^, with improvement or resolution of some of the weight-related co-morbidities [5,6]. The LSG is a relatively safe procedure with a morbidity rate of 0 to 17.5% and an overall mortality rate of 0 to 1.2%, which is less than the comparable Roux-en-Y Gastric bypass, another commonly performed bariatric surgery, and thus should be available to patients who may benefit from this intervention [7,8].

## Case presentation

This is a case of a 59-year-old morbidly obese female with situs inversus totalis who presented for a laparoscopic sleeve gastrectomy. Her BMI was 38 (height 4 ft 11.5 inches, weight 188.2 pounds), and she had a lifelong history of morbid obesity and obesity-related comorbidities, including obstructive sleep apnea requiring a continuous positive airway pressure machine, an elevated hemoglobin A1c (5.8) and a fasting blood glucose increasing her risk of developing diabetes mellitus, and degenerative joint disease which significantly impacted her ability to exercise. The main challenges she identified in losing weight involved eating carbohydrate rich foods, overeating during meals and limited activity due to musculoskeletal pain. The patient had made multiple attempts to lose weight through commercial dieting programs but had been unsuccessful. The patient also completed a six-month medically supervised diet through her primary care provider, which also included working closely with a bariatric registered dietician, following a strict diet of about 1800 calories/day and performing modified exercise, about 120 minutes/week. Despite these intense medical weight loss efforts, she was unable to maintain a healthy weight. The patient was motivated to try bariatric surgery after she witnessed the significant weight loss success her daughters had from this intervention. Her greatest hope from the bariatric surgery was to be healthier and to alleviate her obesity-related comorbidities.

The patient’s surgical history included cesarean section and evacuation of an ectopic pregnancy. She was a former smoker, quitting over 25 years ago, and has no other history of substance or alcohol use. Family history is positive for obesity, diabetes, hypertension, coronary artery disease and hypercholesterolemia. She did not take any medications, including supplements, except for Ibuprofen 800 mg 1–3/daily for musculoskeletal pain.

The patient underwent a comprehensive evaluation and treatment plan prior to the surgery including: psychiatric evaluation and clearance, nutritional consultation with a registered dietician, education about bariatric surgery and pre/post op expectations, routine preoperative labs, UGI, esophagogastroduodenoscopy (EGD), pulmonary function analysis, and a cardiovascular exam. Situs inversus totalis was confirmed with abdominal X-ray and CT, and echocardiogram. Pre-procedural evaluation with UGI revealed mild gastroesophageal reflux observed to the level of the distal one-third esophagus and small sliding-type hiatal hernia. EGD revealed normal esophagus and duodenum. Stomach biopsy was obtained for antral gastritis, and no helicobacter organism was identified. Abdominal ultrasound confirmed fatty liver disease. Pulmonary function analysis including spirometry, lung volumes and diffusion was normal. A pre-procedural cardiovascular exam was performed due to dextrocardia to exclude other structural cardiac abnormalities. 2-D echocardiography with M-mode demonstrated a left ventricular ejection fraction of 60%, no structural abnormalities in the aortic, pulmonic, tricuspid and mitral valve, or right and left atrium and ventricles. Color Doppler and continuous and pulse wave Doppler demonstrated mild pulmonic regurgitation and mild tricuspid regurgitation.

The patient discontinued the Ibuprofen more than one month before her surgery and began a low-calorie liquid diet two weeks prior to surgery. On the day of surgery, preoperative antibiotics, Cefazolin, were given within 60 minutes before the first incision. Prior to induction, the patient received 5000 units of heparin subcutaneously and sequential compression device (SCD) boots were placed for deep vein thrombosis (DVT) prophylaxis. No beta-blockers were administered. The patient was placed in the supine position and general anesthesia was induced. A Foley catheter was placed and the patient was supported with positioning devices including: arms on padded arm boards, gel pad under left axilla, footboard with gel pads, hover mat and a bariatric safety belt over patient’s thighs. The abdomen was then prepped and draped in the standard surgical fashion.

A Veress needle was used in the right upper quadrant to access the abdomen and insufflation was created to 15 mmHg. Veress was removed and replaced with a 5 mm trocar and the scope was placed. Additional trocars were placed in the following position: right and left 5 mm lateral trocars, right and left 12 and 15 mm supra-umbilical trocars. The placement of all retractors and graspers was adjusted accordingly to the mirror image anatomy of the intraabdominal organs. The primary surgeon was positioned on the left side of the patient and the assisting surgeon on right side of the patient. Situs inversus totalis was confirmed with the majority of the liver oriented to the patient’s right, spleen on the right, greater curvature of the stomach on the right and gallbladder on the left.

**Figure 1:**
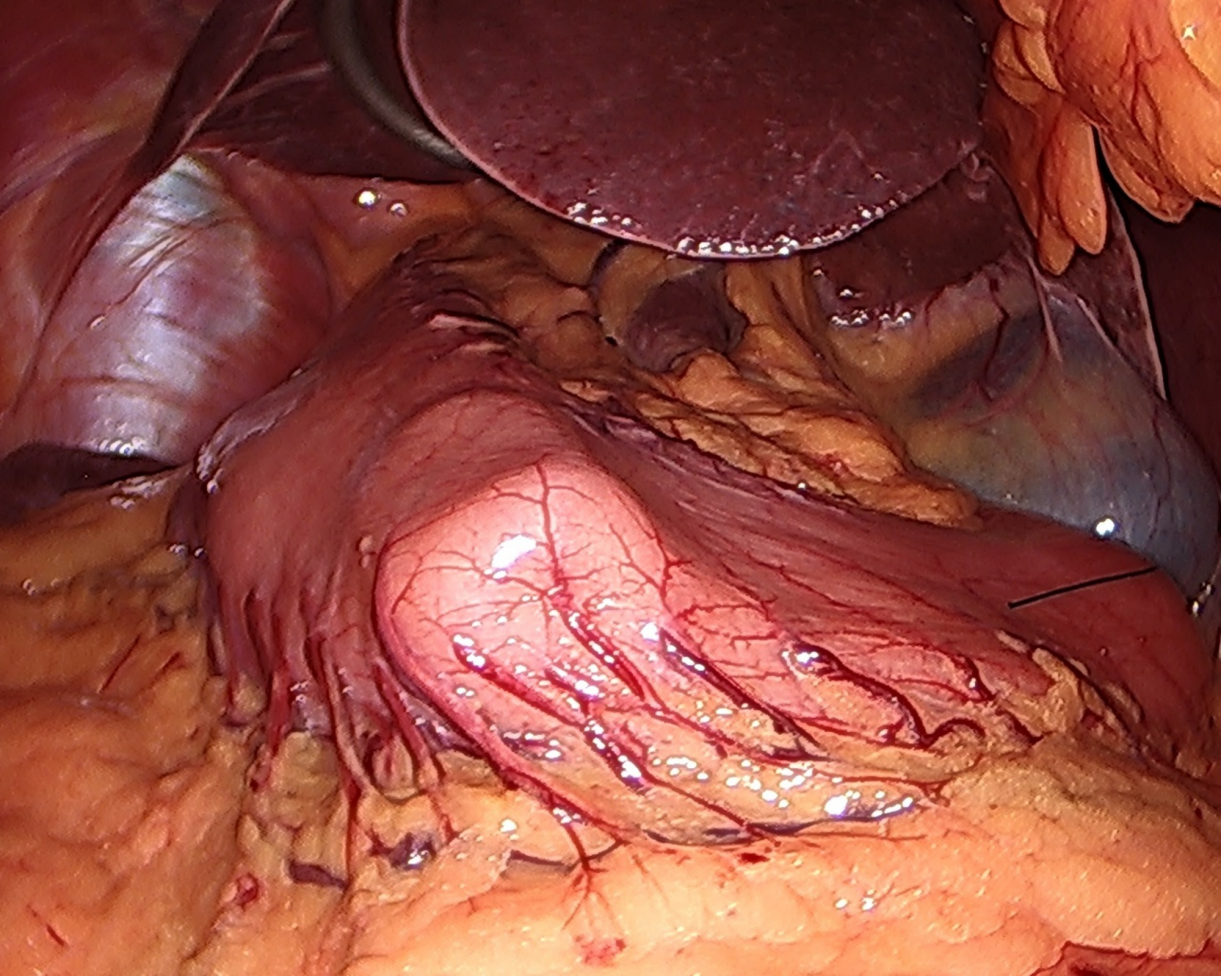
42-French bougie oriented along the lesser curvature of the stomach to identify the antrum. Note the surrounding mirrored anatomy including the liver and gallbladder.

**Figure 2:**
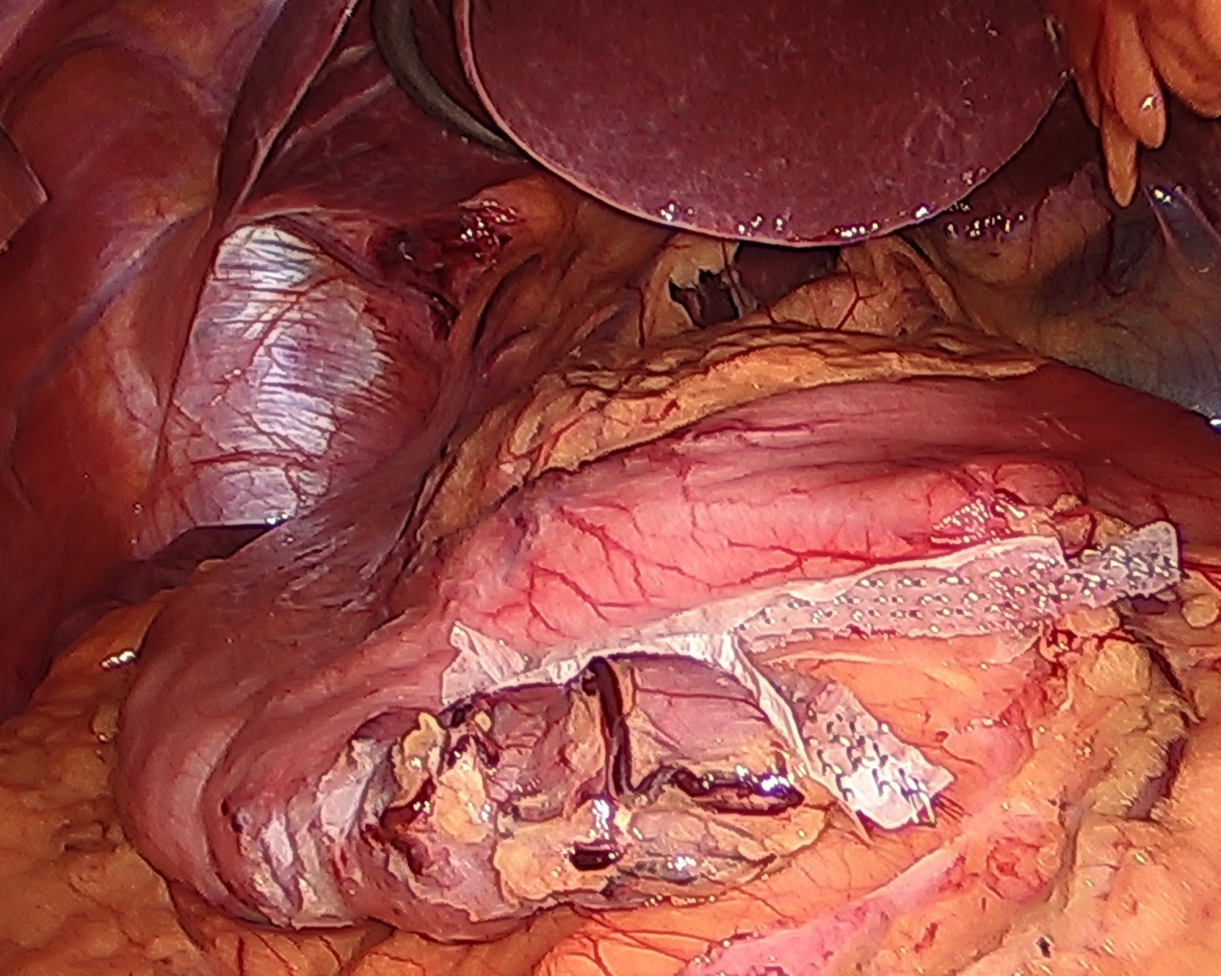
The stomach stapled and divided in a vertical stepwise fashion toward the angle of His using an Ethicon Echelon Flex triple staple line power stapler (Ethicon Inc., NJ, USA).

**Figure 3:**
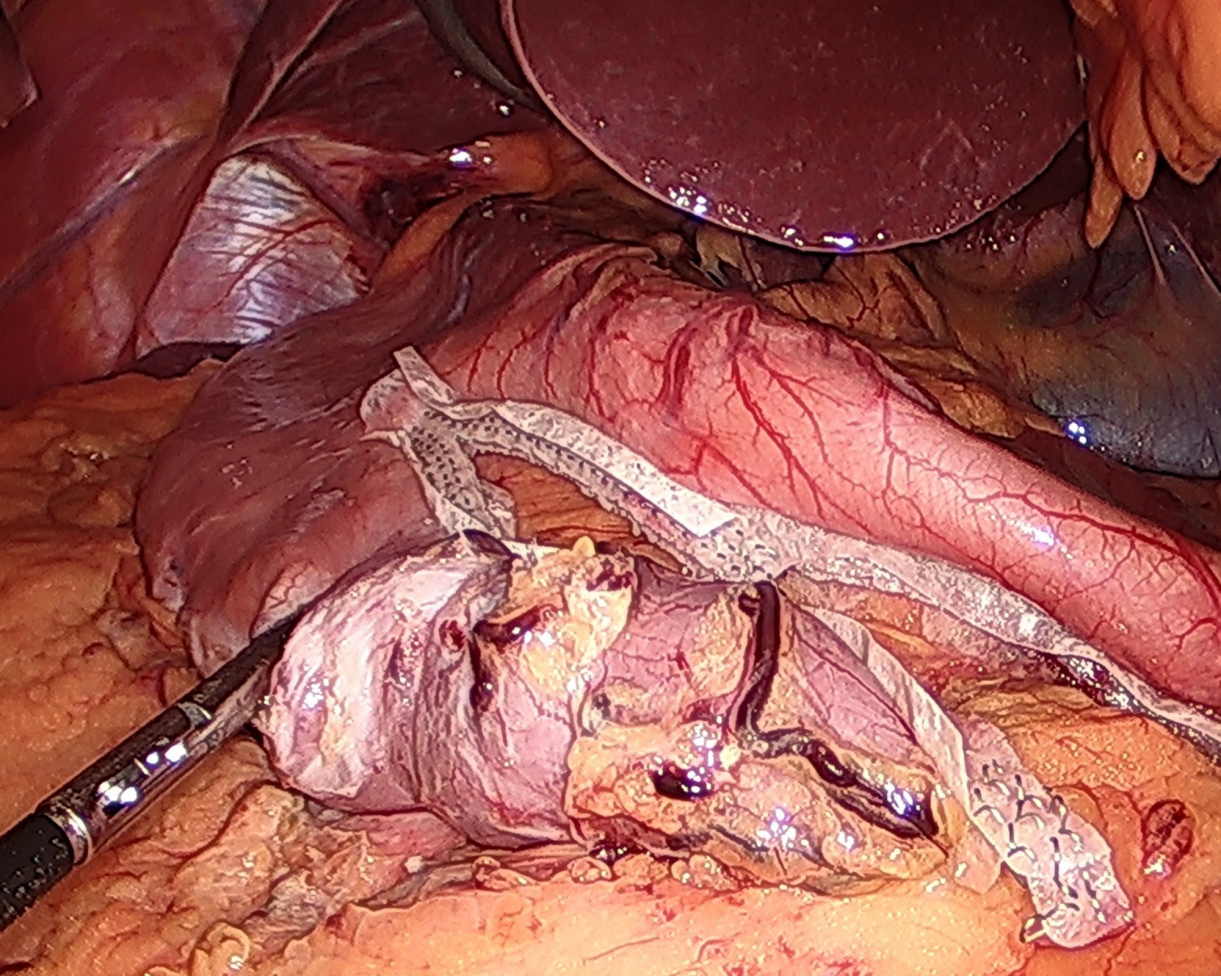
The stomach staple line.

**Figure 4:**
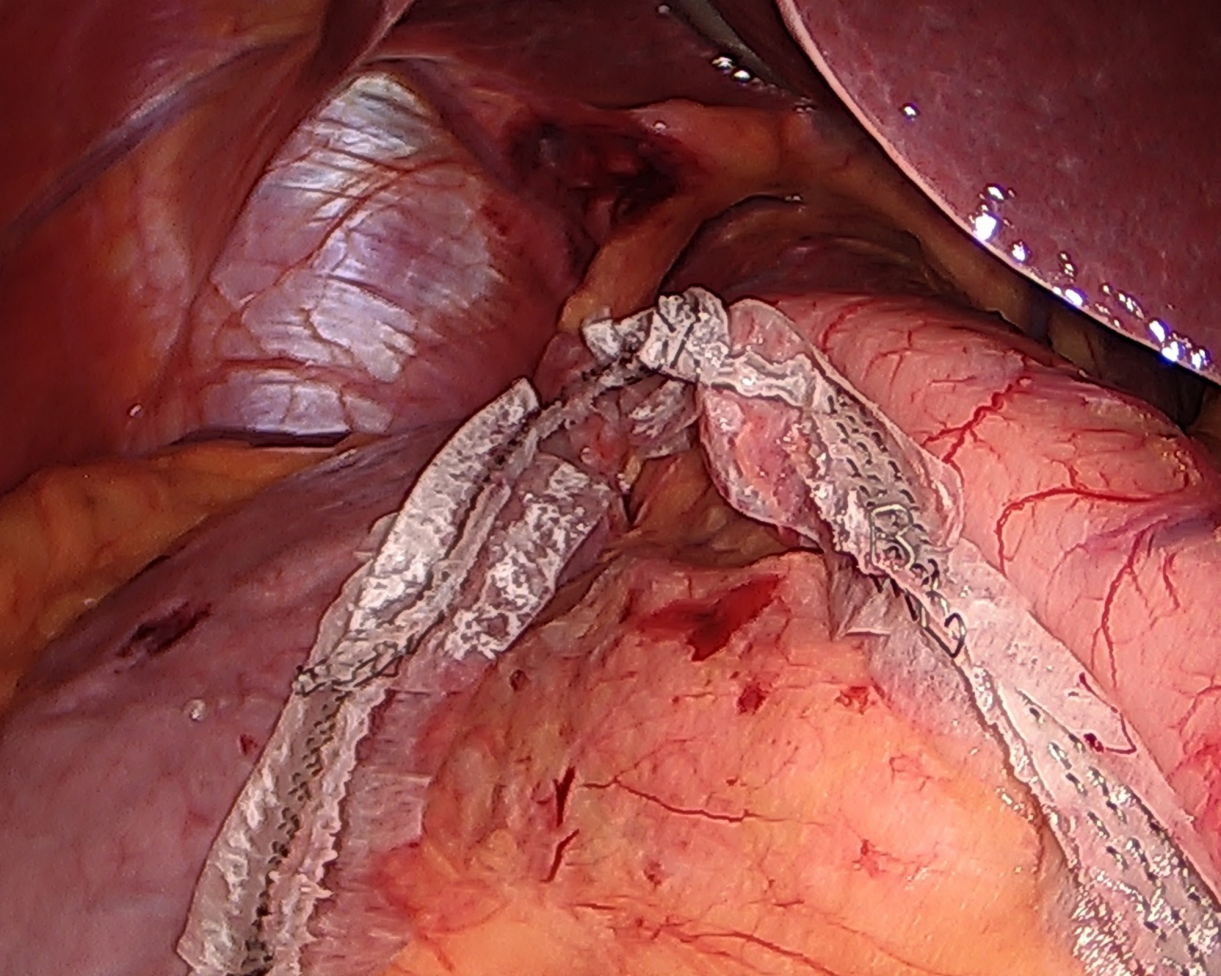
The stomach staple line continued.

The operating table was placed in the reverse Trendelenburg position. The patient’s right-sided half of the liver was retracted cephalically using a Nathanson retractor to expose the vicinity of the esophageal hiatus. The peritoneum over the cardia was incised using the Ethicon Harmonic scalpel, and the plane between the cardia and the left crus of the diaphragm was opened to expose the right diaphragmatic crus. No hiatal hernia was present. A point 5 cm proximal to the pylorus along the greater curvature of the stomach was marked corresponding to the incisura angularis just proximal to the crow’s foot of Latarjet’s nerve. The vessels along the greater curvature and all the short gastric vessels were sealed and divided using the Ethicon Harmonic scalpel, freeing the greater curvature and the fundus of the stomach. A 42-French bougie was placed and oriented towards the antrum along the lesser curvature (Figure 1). The stomach was stapled and divided alongside the tube in a vertical fashion towards the angle of His (Figures 2-4).

**Figure 5:**
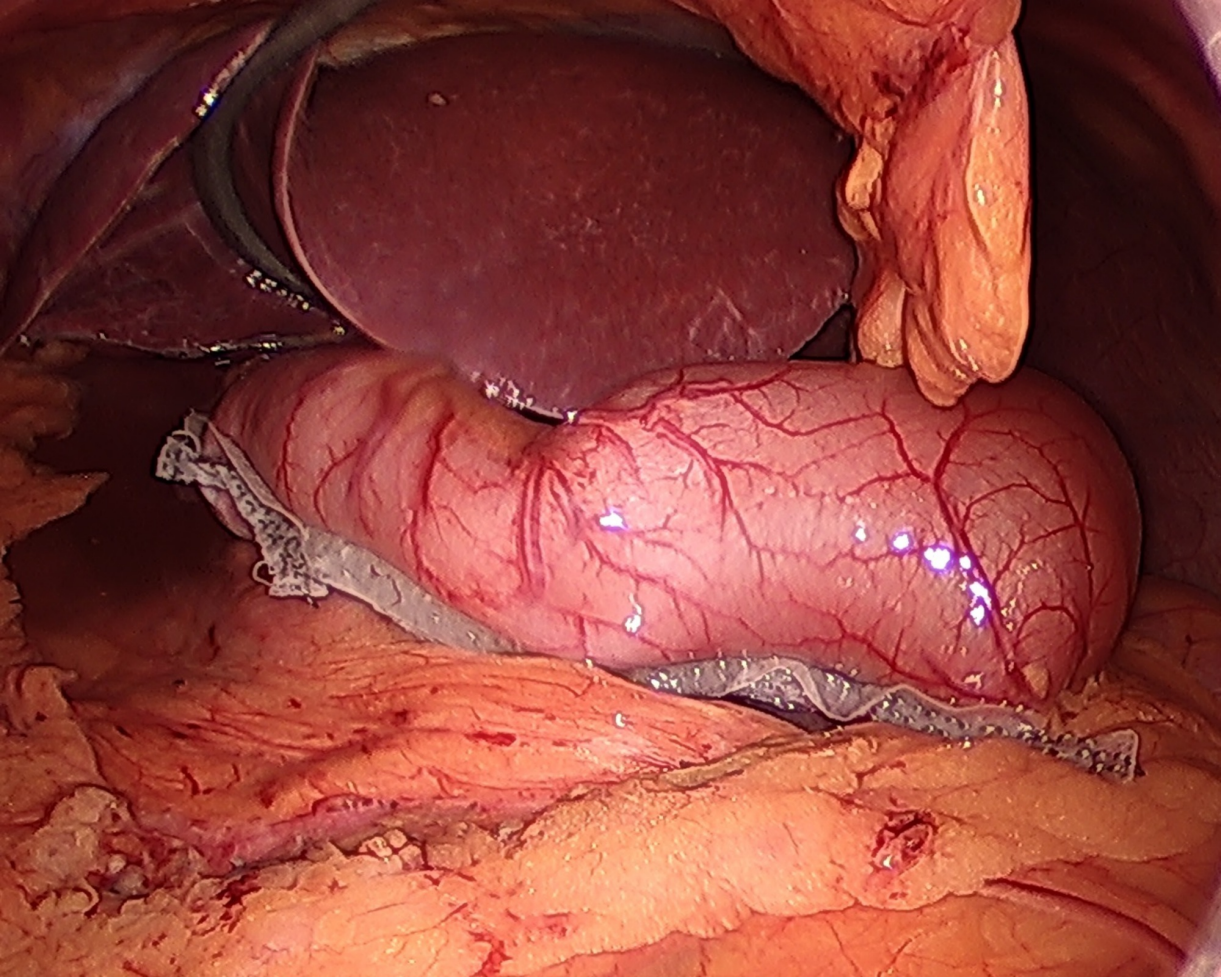
Hemostasis at the external staple line.

**Figure 6:**
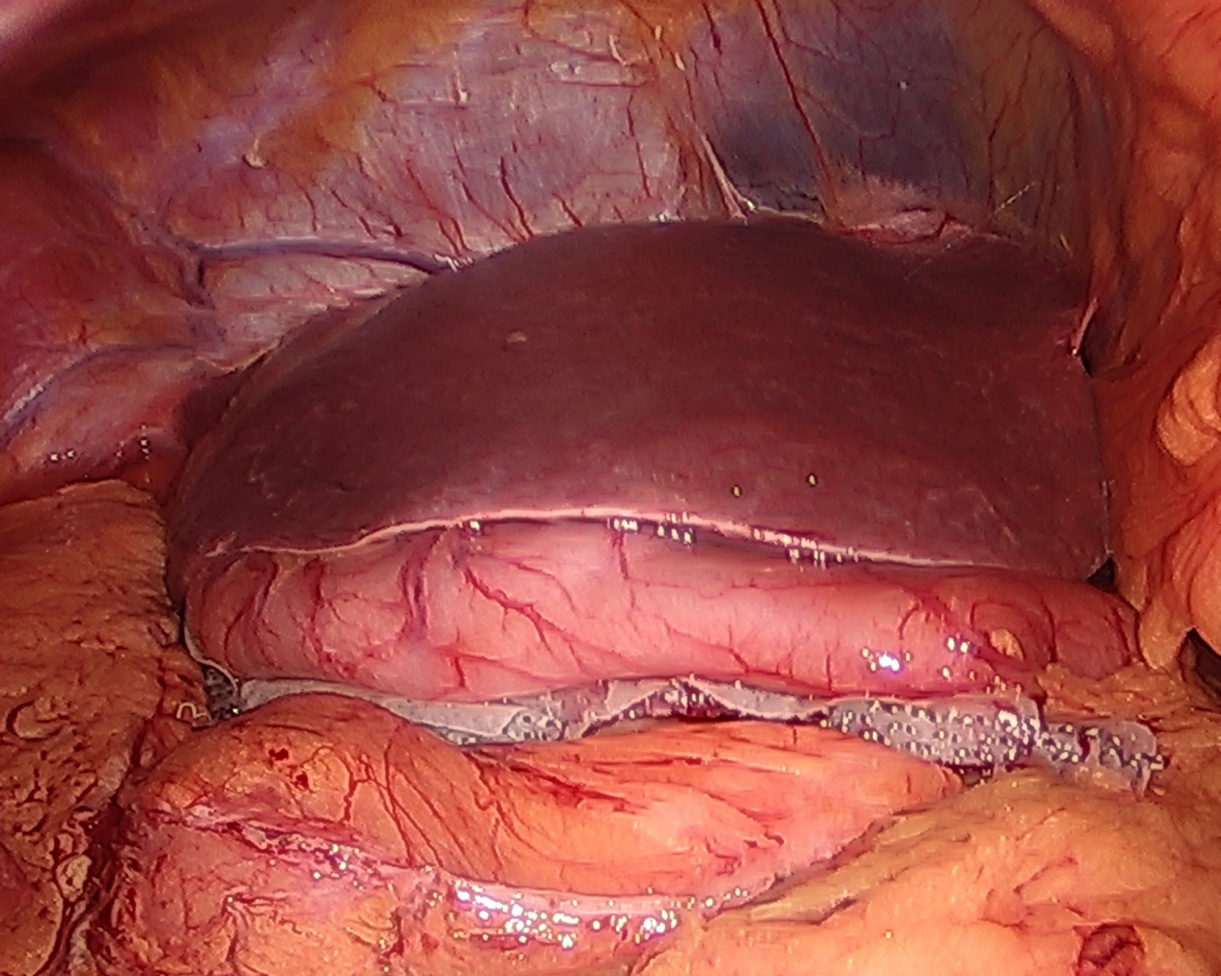
View of abdominal cavity in situs inversus totalis patient after the laparoscopic sleeve gastrectomy is complete.

**Figure 7:**
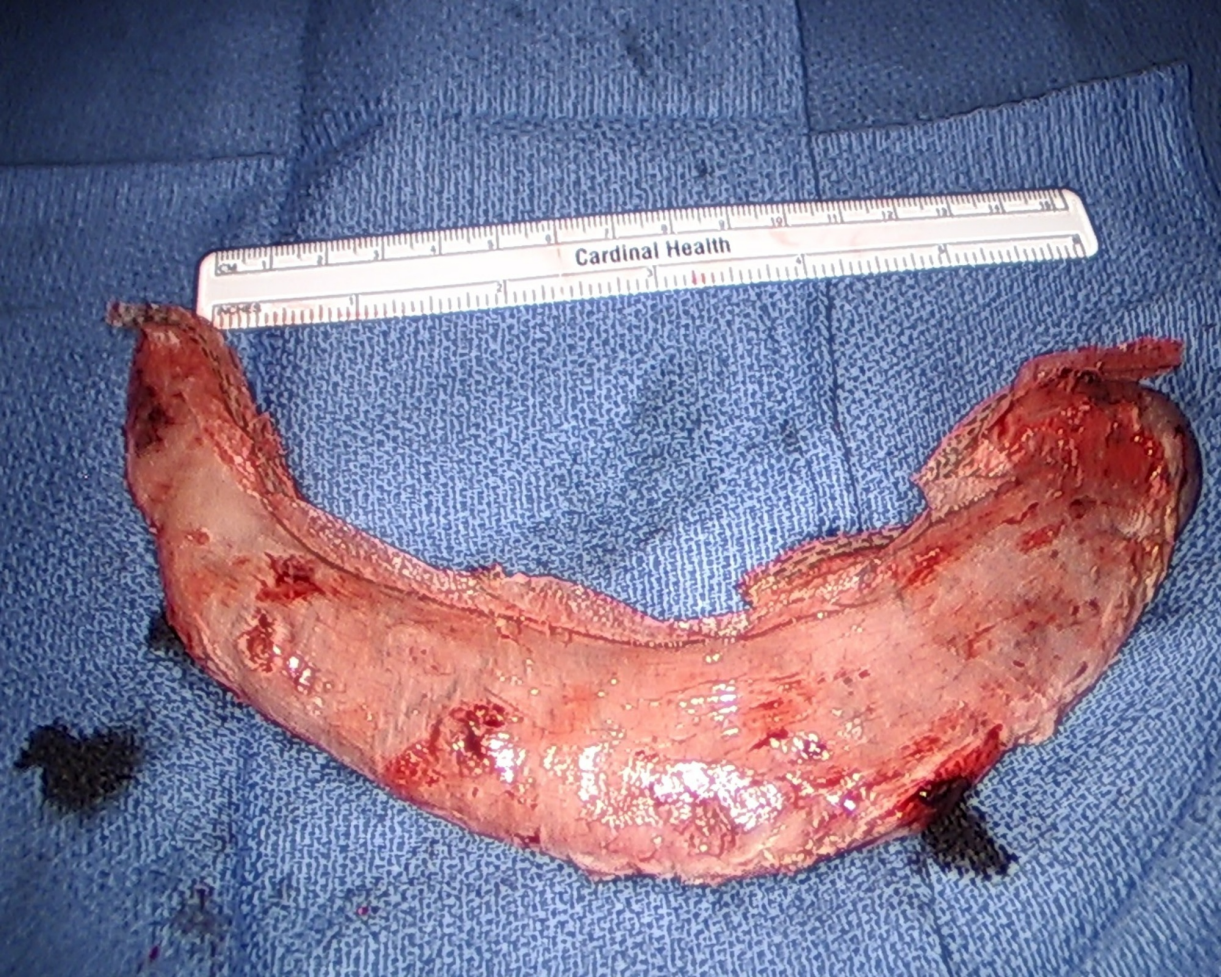
Resected greater curvature and fundus of the stomach.

An Ethicon Echelon Flex triple staple line power stapler with a total of one black, three green, and one gold staple loads was used. All staple loads were 60 millimeters in length with staple line bio-absorbable reinforcement. Hemostasis at the external staple line was achieved (Figure 5). The stomach was removed from the abdomen via the left-sided 15 mm trocar (Figures 6, 7). Intraoperative endoscopy was performed using a 5 mm Olympus Ultrathin gastroscope revealing no areas of stenosis, internal staple line bleeding, nor staple malformation or leak seen. Total operation time was 108 minutes. There were no post-operative complications.

Post-operative care included pain management and DVT prophylaxis with SCD, heparin and early ambulation four hours post-surgery. A UGI was performed on post-op day 2 with normal findings for a post-bariatric surgery evaluation and clear fluids were started. The patient was discharged on post-op day 3 without any complications. Upon discharge, the patient had a treatment plan that was previously discussed that included instructions for diet and medications, including vitamin supplementation and antacids. At her three-month follow-up, the patient was still without complications and very pleased with her weight loss of 46.2 lbs, weighing 142 lbs (BMI 29).

## Discussion

Few cases of laparoscopic surgery in situs inversus patients have been reported in the literature. The limited cases of patients with situs inversus undergoing laparoscopic gastric banding, Roux-en-Y Gastric bypass, and gastric sleeve that have been reported demonstrate no preferred or standardized approach to the positioning of the surgeon and the instruments during these surgeries [9-11]. One approach that has been used during laparoscopic gastric sleeves is placing the patient in the French position, where the patient’s thighs are abducted with the surgeon positioned between the legs [11, 12]. One case study by Catheline et al. utilized this approach and reported the surgeon having difficulty in using the right hand in place of the left hand during this procedure [11]. Alternatively, the laparoscopic Roux-en-Y Gastric bypass has been performed using the mirror image approach, as in our case, where the entire procedure is performed in the reverse fashion [10,12]. The case reported by Wittgrove and Clark found it challenging to manipulate instruments from the opposite side of the table because most of the tasks were then performed by the opposite (non-dominant) hand [12]. Manipulating the foot controls, which in their case ran the electrocautery unit, proved to be challenging as well. Unlike the primary surgeon who had experience as being both primary and an assistant, the assisting surgeon only had experience in assisting and was not accustomed to operating laparoscopically with the right hand. Nonetheless, the primary surgeon still reported difficulty with using chiefly the opposite hand. In the case study by Ahmed and O'Malley utilizing this reverse approach, the major challenge reported was allowing time in the beginning of the procedure to familiarize oneself with the mirror anatomy [10]. They reported no alteration of the surgeon’s use of his dominant right hand, and the operating time was only slightly longer (160 mins vs 105 mins) [10].

The additional operating time to perform a bariatric surgery on a situs inversus patient has also been reviewed. In a literature review of bariatric surgery in situs inversus patients Aziret et al. included a comparison of operating time between two laparoscopic gastric sleeve cases [13]. One having an operating time of 61 minutes compared to another 105 minutes. The increase in time, however, was attributed to adhesions from a previous operation (open cholecystectomy) and not the change in anatomy. This suggests that operating on a patient with a previous abdominal surgery could possibly be a stronger indication of prolonged operating time than is situs inversus.

Our case was a laparoscopic sleeve gastrectomy procedure using the mirror image approach. The primary surgeon was positioned on the patient’s left and the assistant on the patient’s right, with complete reversal of all the instruments. While there was no change in handedness, however, challenges did include adapting to the variation in anatomy and manipulation of instruments. The surgeon addressed the variation in anatomy that may have caused confusion during the surgery by closely studying pre-operative imaging of the patient. Additionally, it was noted that during the operation, it was difficult to capture the right side of the liver cephalically with the Nathanson liver retractor due to the shape at the end of the retractor. Furthermore, the ability to use the right rib to help bend the staple into the proper position for the staple line was difficult. The operation time reported for this case was 108 minutes, compared to the average 64 minutes of operation time the surgeon utilizes to perform a laparoscopic sleeve gastrectomy and endoscopy in uncomplicated cases. Despite the added time and complexity to the case, performing the laparoscopic sleeve gastrectomy with a mirror image approach proved to be successful.

## Conclusions

Pre-operative evaluation should be performed in all patients with situs inversus to screen for other common abnormalities in this patient population that may pose operative risks, such as respiratory, cardiovascular, and digestive anomalies. In the absence of high-risk abnormalities, morbidly obese patients with situs inversus can safely be offered bariatric surgery. The laparoscopic sleeve gastrectomy using the mirror image approach has shown to be safe and effective in patients with situs inversus totalis.
